# Viscosity of hcp iron at Earth’s inner core conditions from density functional theory

**DOI:** 10.1038/s41598-020-63166-6

**Published:** 2020-04-14

**Authors:** Sebastian Ritterbex, Taku Tsuchiya

**Affiliations:** Geodynamics Research Center, Ehime University, 2-5 Bunkyo-cho, Matsuyama, 790-8577 Japan

**Keywords:** Planetary science, Solid Earth sciences

## Abstract

The inner core, extending to 1,221 km above the Earth’s center at pressures between 329 and 364 GPa, is primarily composed of solid iron. Its rheological properties influence both the Earth’s rotation and deformation of the inner core which is a potential source of the observed seismic anisotropy. However, the rheology of the inner core is poorly understood. We propose a mineral physics approach based on the density functional theory to infer the viscosity of hexagonal close packed (hcp) iron at the inner core pressure (*P*) and temperature (*T*). As plastic deformation is rate-limited by atomic diffusion under the extreme conditions of the Earth’s center, we quantify self-diffusion in iron non-empirically. The results are applied to model steady-state creep of hcp iron. Here, we show that dislocation creep is a key mechanism driving deformation of hcp iron at inner core conditions. The associated viscosity agrees well with the estimates from geophysical observations supporting that the inner core is significantly less viscous than the Earth’s mantle. Such low viscosity rules out inner core translation, with melting on one side and solidification on the opposite, but allows for the occurrence of the seismically observed fluctuations in inner core differential rotation.

## Introduction

The dynamical processes of the inner core rely significantly on the viscous strength of iron^1,2^. Since plastic deformation of iron may produce crystallographic preferred orientations^3^ (CPO), creep is commonly considered to be a potential source contributing to the seismic anisotropy observed in the inner core^4^. The viscosity of the inner core also influences the rotational dynamics of the Earth^5^. In spite of its relevance to the Earth’s dynamics, the viscosity of the inner core is poorly constrained. Estimates from geophysical observations of Earth’s nutation^6^ predict inner core viscosities around ~2–7 × 10^14^ Pa s, while values of ~10^17^ Pa s are suggested from observations of polar wander^5^. An upper bound value of 3 × 10^17^ Pa s was inferred from geodynamic modeling of the gravitational coupling between the core and mantle^7^ in accordance with seismic observations of fluctuations in the rate of the inner core differential rotation. Viscoelastic relaxation experiments^8^ of iron report a viscosity of ~10^13^ Pa s at ambient pressure, but higher pressure and larger grains in the inner core^9^ tend to increase viscous strength. Experimental approaches however require significant extrapolation of transport and flow properties in metals to the inner core condition due to technical difficulties, leading to a wide uncertainty (~10^11–22^ Pa s)^10–12^. On the other hand, recent simulations^13^ predict a viscosity comparable to that of liquid iron in the outer core close to ~10 mPa s, based however on results questioned by Schultz *et al*.^14^ and inconsistent with observations of normal modes involving the inner core (*e.g*. Mäkinen & Deuss^15^), suggesting it to behave as a solid.

Although the stable phase of iron in the inner core is still matter of debate, most experimental studies suggest the hcp phase to be the likely candidate (*e.g*. Tateno *et al*.^16^; Anzellini *et al*.^17^). Its viscosity depends on the dominant creep mechanism governing its deformation. Creep rates in metals at *T* > 0.4*T*_*m*_ are strongly controlled by bulk self-diffusion accommodated by the migration of atomic vacancies^18^. Here, we quantify the self-diffusion in hcp iron explicitly, computing all required parameters by the first-principles density functional approach, which is a powerful tool to derive lattice defect properties of Earth materials (*e.g*. Ritterbex *et al*.^19^) because of its non-empirical description of atomic bonding. Particularly at the relevant inner core conditions, there is currently no other way to obtain diffusional and rheological properties of iron.

## Results

### Iron self-diffusion

Iron in the solid inner core is stable at pressures between ~329 and 364 GPa and temperatures of 5,000–7,000 K^16,20^. We predict atomic diffusivity of iron at those *P*,*T* in the framework of transition state theory^21^ (TST) as previously developed and applied to metals^22^. Self-diffusion in metals occurs typically in the intrinsic regime by vacancy migration^23^. The associated self-diffusion coefficient *D*_*sd*_ can be represented as^22,23^

1 $${D}_{sd}=f{Z}_{f}\frac{{Z}_{m}}{6}{l}^{2}{X}{\Gamma },$$

where *f* is the correlation factor to account for possible unsuccessful or backward atomic jumps^23^, *Z*_*f*_ the number of equivalent ways to form a vacancy, *Z*_*m*_ the number of equivalent migration paths, *l* the jump distance, Γ the atomic jump frequency and *X* the equilibrium point defect concentration given by

2 $$X=\exp \left(\frac{\Delta {S}_{f}}{{k}_{b}}\right)\exp \left(-\frac{\Delta {H}_{f}}{{k}_{b}T}\right),$$

where *k*_*b*_ is the Boltzmann constant, and Δ*H*_*f*_ and Δ*S*_*f*_ are the enthalpy and entropy of vacancy formation, respectively. According to TST, the jump frequency $\varGamma ={\nu }^{\ast }\exp \left(-\frac{\Delta {H}_{m}}{{k}_{b}T}\right)$ is represented in terms of the attempt frequency v* and the migration enthalpy Δ*H*_*m*_. All these parameters required are evaluated by first-principles total energy calculations, lattice dynamics and electronic structure theory calculations (see Methods).

**Figure 1:**
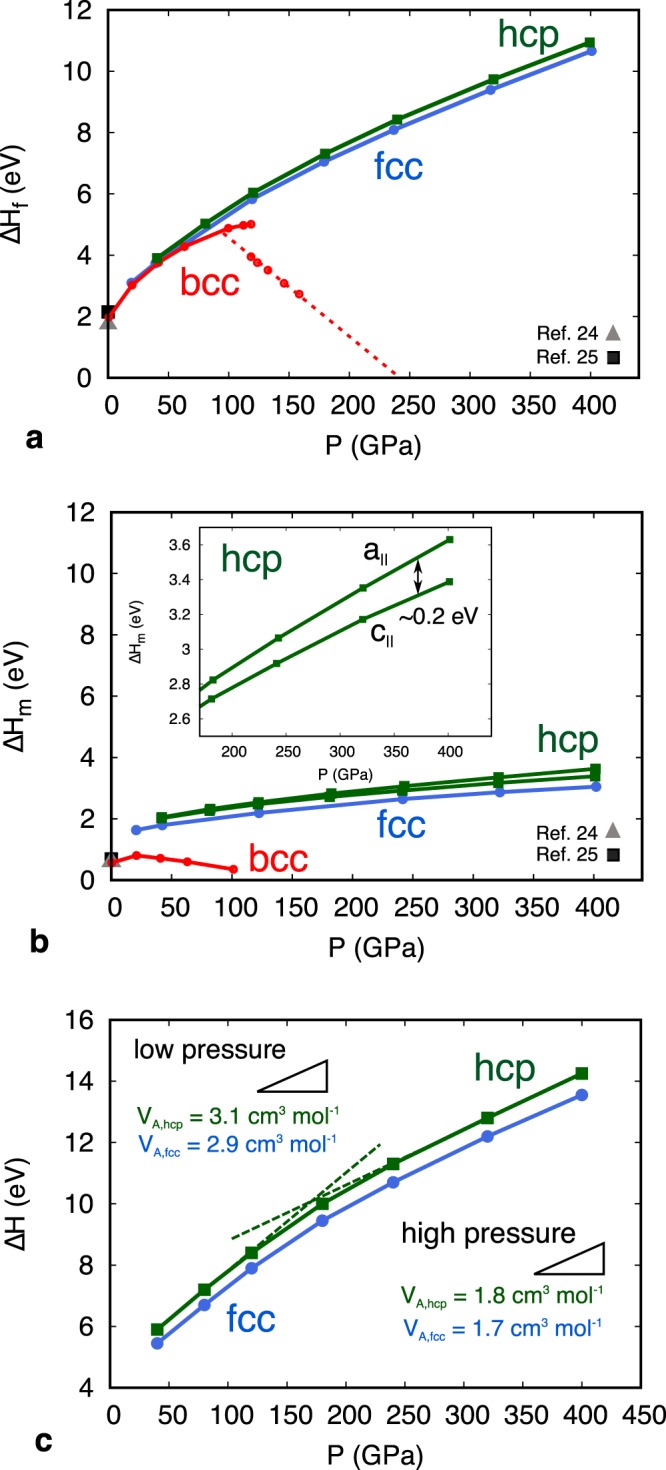
Defect energetics as a function of pressure from first-principles calculations. (**a**) Vacancy formation enthalpy at static temperature for bcc (red), fcc (blue) and hcp (green) iron. The red dotted line represents extrapolation of the vacancy formation enthalpy of bcc iron at high pressure. A formation enthalpy of 0 eV is expected at ~240 GPa, comparable to the pressure corresponding to the tetragonal shear instability of bcc iron^26^. (**b**) Vacancy migration enthalpy of bcc (red), fcc (blue) and hcp (green) iron at static temperature. The inset shows the migration enthalpies of in-basal $({{\rm{a}}}_{\parallel })$ and out-basal $({{\rm{c}}}_{\parallel })$ plane migration in hcp Fe. (**c**) The activation energy Δ*H* = Δ*H*_*f*_ + Δ*H*_*m*_ of self-diffusion in fcc (blue) and hcp (green) Fe at static temperature. The slope corresponds to the activation volume ${V}_{A}=\partial H/\partial P$ for vacancy diffusion. The *V*_*A*_ within the pressure regimes 120 > *P* > 240 GPa are indicated where Δ*H* varies almost linearly with *P*. In those regimes, the *V*_*A*_ is obtained by a least-squares regression of our data.

The formation (Δ*H*_*f*_) and migration (Δ*H*_*m*_) enthalpies of hcp, face centered cubic (fcc) and body centered cubic (bcc) iron are computed as a function of pressure (Fig. 1a,b). The results for bcc Fe at ambient pressure are in good agreement with previous computational studies^24,25^. Formation enthalpies of hcp and fcc Fe increase monotonously with increasing pressure until ~400 GPa, whereas that of bcc Fe starts decreasing at ~120 GPa (Fig. 1a). Similarly, migration enthalpies of hcp and fcc Fe increase monotonously with pressure, whereas that of bcc Fe starts decreasing over ~20 GPa (Fig. 1b). The anomalous pressure dependency found in bcc Fe results from the tetragonal shear instability at high pressure^26^. Recent molecular dynamics simulations^14,20^ (MD) demonstrate that pure bcc Fe at inner core pressures remains mechanically unstable up to ~7,000 K and predict that the close-packed structure of pure iron is more stable at inner core conditions. Moreover, our results suggest that the presence of vacancies enhances the mechanical instability of bcc Fe at high pressure. Therefore, we focus on the close-packed structures as the likely polymorph of iron stable in the inner core. Interstitial defects in the close-packed phases of Fe are found to be energetically unfavorable with larger formation enthalpies of ~3.5 eV than those of vacancies at inner core pressure, implying that vacancies are more abundant (Eq. 2) and that self-diffusion is mainly controlled by the diffusion of vacancies.

Vacancy migration enthalpies are determined by structure relaxation of equilibrium and transition states (see Methods). Results of transition states in hcp Fe are additionally verified by the climbing image nudged elastic band approach^27^ (CI-NEB) (see Methods and Supplementary Information). Atomic migrations in bcc and fcc Fe are unique and occur along the <111> and <110> directions, respectively, whereas in-basal (parallel to *a*) and out-basal plane (along *c*) diffusion are possible in hcp Fe. The energy barrier of out-basal plane diffusion at 320 GPa from structure relaxation (3.17 eV) is in good agreement with the one obtained by the CI-NEB approach (3.29 eV) (Supplementary Fig. 1). Figure 1b shows that atomic diffusion in hcp Fe becomes slightly anisotropic at higher pressures with a difference in Δ*H*_*m*_ up to ~0.2 eV at 360 GPa in favor of out-basal plane diffusion, reaching ~6% of the total migration enthalpy at 360 GPa. Since the lowest energy diffusion path is most favorable, self-diffusion in hcp Fe is considered to occur more easily through the out-basal plane path.

The activation volumes for self-diffusion ${V}_{A}=\partial H/\partial P$ are found to decrease significantly at larger compressions in the close-packed phases of iron (Fig. 1c). Previous experiments^11^ report a constant *V*_*A*_ of 2.6 cm^3^ mol^−1^ for Fe-Ni interdiffusion in an fcc iron alloy up to 60 GPa, in fair agreement with our results. At inner core pressures however, the *V*_*A*_ is significantly smaller and only ~60% of *V*_*A*_ at *P* < 120 GPa. The nearly constant *V*_*A*_ in close-packed iron up to ~120 GPa followed by a significant decrease at larger compression suggests that the self-diffusivities derived at low pressures cannot be extrapolated to the inner core condition by using a constant *V*_*A*_. It is worth mentioning that the magnitude and pressure dependencies of defect energetics in hcp and fcc Fe are comparable (Fig. 1), implying that their self-diffusivities (Eq. 1) might be similar even up to the pressures of the inner core.

**Figure 2:**
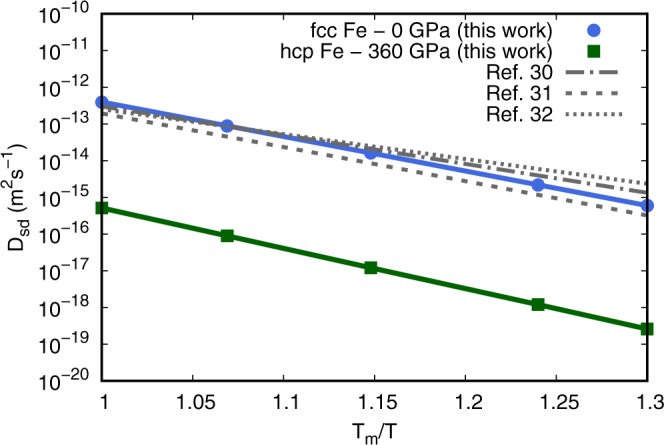
Arrhenius plot of the self-diffusion coefficients of fcc and hcp iron from first-principles calculations*. T* is normalized with *T*_*m*_ = 1,811 K for Fe at ambient pressure (Dorogokupets *et al*.^63^) and with *T*_*m*_ of 6,370 K (Alfè^33^) for hcp Fe at inner core pressure. Calculated results for fcc Fe are compared with experimental results^30–32^.

A combination of lattice dynamics (LD) theory and electronic structure theory are adopted to determine the entropic and vibrational contributions to the diffusion coefficient (Eq. 1) of close-packed Fe in the quasi-harmonic approximation (QHA). These thermodynamic properties are derived from the Helmholtz free energy *F*(*V*, *T*) as

3 $$F(V,T)=E(V)+{F}_{vib}(V,T)+{F}_{el}(V,T)-T{S}_{conf}(V,T)-T{S}_{mag},$$

where *E* is the static energy as a function of volume *V*, *F*_*vib*_ and *F*_*el*_ the vibrational and electronic contributions to the free energy, and *S*_*conf*_ and *S*_*mag*_ the configurational and magnetic entropy, with the latter being only considered for fcc Fe at 0 GPa since nonmagnetic states become energetically favorable with increasing pressure (see Methods). The temperature effects on all diffusion parameters are determined based on the calculated equations of state (EoS). Free energies of defect-free close-packed Fe are computed at five volumes with lattice parameters varying by 1.5% to determine the EoS (Supplementary Fig. 2). Phonon frequencies of all systems are obtained by the direct force constant approach^28^ to determine the contribution of *F*_*vib*_ and the attempt frequencies v* which are estimated from the maximum frequencies of the phonon spectra^29^ (see Supplementary Information). Migration enthalpies Δ*H*_*m*_ and the Gibbs free energies of vacancy formation Δ*G*_*f*_ are calculated corresponding to the equilibrium volumes of close-packed Fe at the *P*,*T* conditions of interest, with Δ*G*_*f*_ defined as

4 $$\Delta {G}_{f}=\left[G(N-1)-G(N)\frac{N-1}{N}\right]=\Delta {H}_{f}-T\Delta {S}_{f},$$

where *G*(*N* − 1) and *G*(*N*) correspond to the Gibbs free energy of a defective and a defect-free system with *N* atoms, respectively and Δ*S*_*f*_ the total entropy of vacancy formation. The Gibbs free energy of vacancy formation Δ*G*_*f*_ in hcp Fe is found to be only ~80% of the formation enthalpy Δ*H*_*f*_ at the inner core temperature (Table 1). This emphasizes on the importance of considering correctly the contribution of Δ*S*_*f*_ to the total Gibbs free energy of vacancy formation at the inner core temperature. To benchmark our computational approach, self-diffusion of fcc Fe is calculated at ambient pressure close to the melting temperature *T*_*m*_ to compare with experimental results. The diffusion coefficients (Eq. 1) of close-packed Fe are obtained using the computed diffusion parameters (Table 1) after applying a thermal pressure correction at each temperature according to the appropriate EoS. Results for fcc Fe at ambient pressure near *T*_*m*_ are in excellent agreement with experimental results^30–32^ (Fig. 2). The latter shows that atomic diffusivity of fcc Fe is well predicted within the QHA even close to *T*_*m*_, indicating negligible higher-order anharmonic effects on the diffusion coefficients other than the QHA level anharmonicity. This was also shown in other metals^22^ and provide support that atomic diffusivity might be well predicted within the QHA at inner core conditions. The melting temperature of hcp Fe at the inner core pressure is still not well constrained and commonly considered between 6,000–7,000 K^33^. Although the temperature at the inner core boundary (ICB) is expected to be lower than the *T*_*m*_ of pure iron due to its depression by alloying with light elements, the *T*_*m*_/*T* ratio of the inner core is commonly considered to be ~1.15–1.05, corresponding to a typical diffusion coefficient of hcp Fe of ~10^−16^–10^−17^ m^2^s^−1^ (Fig. 2).

**Table 1:** Parameters to calculate diffusion coefficients within the TST (Eq. 1).

Parameters	hcp iron (*P* = 360 GPa, *T* = 5,000 K)	fcc iron (*P* = 0 GPa, *T* = 1,800 K)
**Δ*H***_***f***_ **(eV)**	10.3	1.97
**Δ*H***_***m***_ **(eV)**	3.21	1.41
**Δ*S***_***f***_ **(*k***_***b***_**)**	3.26	7.35
v* **(THz)**	21.3	7.34
***f*** ^23^	0.78146	0.78146

### Iron creep

Since bulk diffusion is dominant close to *T*_*m*_, we inferred the contribution of diffusion creep to the plasticity of hcp Fe by Nabarro-Herring creep^34,35^ (see Supplementary Information). The present diffusion parameters of hcp Fe combined with an inner core grain size in the order of meters, estimated by previous work^9^, leads to a high viscosity of ~10^26^ Pa s, ruling out diffusion creep as an efficient strain producing mechanism (Supplementary Fig. 5). Moreover, this mechanism is not able to produce CPO, being incompatible with the presence of a strong seismic anisotropy observed in the inner core^4^. CPO is commonly produced during dislocation creep of iron at high pressure^3^. Near *T*_*m*_, dislocation creep is expected to be climb-controlled since diffusion is strongly facilitated^18^. This together with considerations of large grains^9^ has led to the suggestion that Harper-Dorn creep controls deformation of the inner core^10^, but its mechanism cannot be fully understood from experiments and its existence has been subject to debate^36^. Yet, the rate-limiting creep behavior of metals at *T* > 0.4*T*_*m*_ can be predicted with climb-controlled dislocation creep models proposed by Weertman^37^ and Nabarro^38^. Weertman’s model assumes that the glide velocity (*v*_*g*_) of dislocations is much larger than that of climb (*v*_*c*_) at high homologous temperature close to melting, due to low lattice friction. Its constitutive equation describing viscous flow in the limit of low stress can be derived as (see Methods)

5 $${\dot{\varepsilon }}_{W}=A(\sigma )\frac{{D}_{sd}\mu b}{{k}_{b}T}{\left(\frac{\sigma }{\mu }\right)}^{3},$$

where $\dot{\varepsilon }$ is the strain rate, *σ* the flow stress, *A*(*σ*) a stress dependent dimensionless parameter depending on the climb distance *d* between glide planes, *b* the modulus of the Burgers vector and *μ* the shear modulus. If, however glide would be slower than climb (*v*_*g*_ < *v*_*c*_) in hcp Fe at the inner core *P,T*, plastic strain may be produced exclusively by pure climb as proposed by Nabarro^38^, in contrast to Weertman creep where strain is mainly produced by glide. This mechanism, known as pure climb creep, is described by the following constitutive equation^38^

6 $${\dot{\varepsilon }}_{N}=\frac{1}{\pi \,{\rm{l}}{\rm{n}}\left(\frac{4\mu }{\pi \sigma }\right)}\frac{{D}_{sd}\mu b}{{k}_{b}T}{\left(\frac{\sigma }{\mu }\right)}^{3},$$

**Figure 3:**
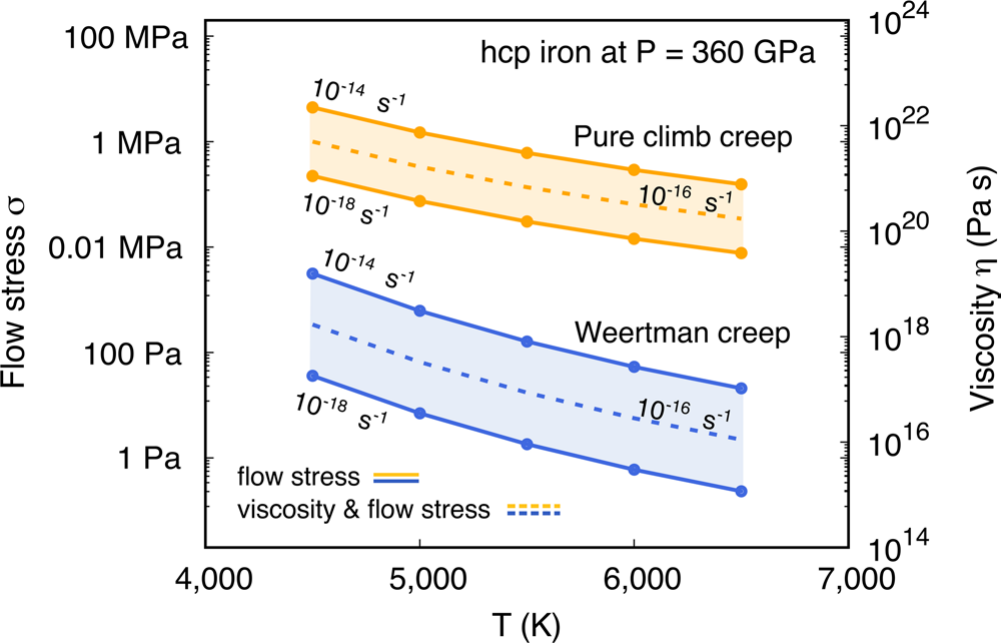
Deformation mechanism map of hcp Fe at 360 GPa containing the limiting cases of climb-controlled dislocation creep (Weertman creep^37^ and pure climb creep^38^). Solid lines correspond to the flow stress σ indicated at the left vertical axis whereas the dotted lines correspond to both the viscosity scale *η* on the right vertical axis and the flow stress σ on the left vertical axis.

The computed diffusion parameters of hcp Fe are used to parametrize the constitutive Eqs. 5 and 6 at the inner core *P*,*T* and a range of relevant steady-state strain rates between 10^−14^–10^−18^ s^−1^, typical for potential inner core convection processes^1^. We employ a shear modulus *μ* = 212 GPa of hcp Fe^39^ and assume basal slip to dominate glide in hcp Fe^3^, *i.e. b* = a and *d* = *c*/2. Results are presented in a deformation mechanism map (Fig. 3). At the inner core temperature ~5,500 K, Weertman creep is the most efficient mechanism operating at typical stresses ~1–100 Pa compared to ~0.01–0.1 MPa required to activate pure climb creep. The associated viscosities *η* are determined as $\sigma /2\dot{\varepsilon }$ and correspond to ~10^16^–10^18^ and ~10^19^–10^22^ Pa s for Weertman and pure climb creep, respectively. The key unknown is the lattice friction opposed to dislocation glide in hcp Fe at inner core conditions. Commonly, lattice friction in metals at *T*_*m*_/*T* ~ 1.1 is low so that ${v}_{g}\gg {v}_{c}$, activating Weertman creep^37^. Also, in absence of lattice friction, mobile dislocations can glide freely under the action of seismic stress and induce seismic attenuation^8^. Indeed, normal mode studies provide evidence of substantial seismic attenuation in the inner core^15^, arguing for low lattice friction of Fe close to *T*_*m*_. Moreover, recent deformation experiments^12^ of hcp Fe inferred that stresses of ~10 Pa are required to activate glide at low strain rates ($\dot{\varepsilon } \sim {10}^{-18}$ s^−1^) and inner core *P*,*T*. This is comparable to the stress needed for Weertman creep to operate (Fig. 3) and provide support for sufficiently low lattice friction in hcp Fe to activate dislocation creep. It is therefore likely that Weertman creep governs plastic flow of hcp Fe in the inner core, unless glide would be hampered by a limited availability of slip systems (*i.e*. not satisfying the von Mises’ criterion)^40^ and deformation is forced to occur by pure climb creep, leading to a significantly more viscous inner core (Fig. 3). Alternatively, twinning or grain boundary sliding (GBS) may ensure plastic flow in hcp Fe if the von Mises’ criterion is not satisfied^40,41^. Those mechanisms rely on intracrystalline plasticity as dislocation creep to maintain macroscopic continuity. This also supports that Weertman creep might play a rate-controlling role in the plasticity of hcp Fe at inner core conditions leading to a viscosity of ~10^17±1^ Pa s (Fig. 3).

## Discussion on the dynamics of Earth’s inner core

Our findings support geophysical estimates^5,6^ of an inner core which is significantly less viscous than the mantle (~10^21^–10^24^ Pa s)^42^, but substantially more viscous than the outer core (~10 mPa s)^13^. The relatively low viscosity of ~10^17±1^ Pa s of hcp iron at inner core conditions inferred from our mineral physics approach is in line with the recent seismic observations of *J*-waves which also point towards a readily deforming inner core^43^. However, the results presented correspond to the viscosity of pure hcp Fe, neglecting the discrepancy between the observed inner core density and that of hcp Fe at the appropriate conditions^44^. This discrepancy can be explained by the presence of a small amount of melt^44^ or by the stabilization of bcc Fe due to alloying with some light elements such as Si^26^. The bcc phase of Fe is expected to be less viscous than the hcp phase because of tetragonal shear weakening at inner core pressure^26^. Thus, the potential presence of melt or bcc Fe might lead to a decrease in viscous strength with respect to pure hcp Fe. This implies that the inner core could be even less viscous than ~10^17±1^ Pa s. In addition, alloying Fe with light elements might influence its mechanical strength by affecting dislocation multiplication and annihilation processes through changes in the glide and climb mobilities, although this is not well understood yet under the relevant *P*,*T* and extremely low strain rate conditions of the inner core. Nevertheless, the inferred inner core viscosity fairly agrees with estimates from geodetic observations^5^. Furthermore, the inner core viscosity is a crucial parameter determining the rotational dynamics of the inner core. Although it has been shown that a steady rate of inner core super-rotation should be negligibly small^45^, the inner core is expected to undergo fluctuations in its rotation rate with amplitudes of 0.1–1° yr^−1^ at timescales of decades to a century^46^. To ensure that the gravitational torque exerted on the mantle by an oscillating inner core does not exceed the observed length-of-day changes, it is required that $\Gamma \,{\tau }\lesssim 2\times {10}^{20}$ N m yr, where Γ is a measure of the gravitational strength between the mantle and the inner core and *τ* the viscous relaxation time of the inner core^47^. An upper bound value of *τ* between 1–6 yr is found, based on the latest estimates of Γ from geodynamic modeling^7^, corresponding to an inner core viscosity of 0.5 − 3 × 10^17^ Pa s^48^, which falls in the range of values derived from our mineral physics approach. An inner core, gravitationally coupled with the mantle, which is much stiffer or weaker than ~10^17^ Pa s would generate larger fluctuations in the rate of inner core rotation than those observed. Our inferred viscosities are thus consistent with findings of the seismically observed small fluctuations in the inner core rotation rate.

Previous geodynamic modeling^2,49^ show that the viscosity derived from our approach might be too low to account for inner core translation, which is one of the hypotheses to explain the hemispherical patterns of seismic anisotropy in the inner core^50^. Instead, if the viscosity of the inner core is lower than ~3 × 10^18^ Pa s, these modeling predict that its dynamics is rather governed by large scale convection. Indeed, our modeling predicts that stresses of tens of Pa are required to deform hcp Fe by Weertman creep at low steady-state strain rates (~10^−16^ s^−1^) which are comparable to the potential driving forces required to initiate various candidates of inner core convection^1,51,52^ supporting that dislocation creep might be a dominant deformation mechanism governing the dynamics of the Earth’s inner core. Since dislocation creep leads to the formation of CPO in hcp metals^3^, it can be expected that plastic deformation of hcp Fe contributes to the observed seismic anisotropy in the inner core. It is finally worth mentioning that dislocation creep exhibits a non-Newtonian rheology which might be important to consider in future geodynamic modeling of the inner core dynamics.

## Methods

### First-principles electronic structure calculations

Our computation method relies on first-principles density functional techniques with the generalized gradient approximation (GGA) applied for the exchange-correlation energy^53,54^. Static relaxations of all structure models were performed based on the Plane-Wave Self-Consistent Field code with the planewave and pseudopotential methods implemented in the Quantum ESPRESSO package^55^. Ultrasoft pseudopotentials^56^ are used to describe the effective core potential of Fe with electronic configurations of 3s^2^3p^6^3d^6.5^4s^1^4p^0^. Pseudo-wavefunction and valence electron density are represented by the planewave basis set with a cutoff energy of 50 Ry. We further apply the Fermi-Dirac smearing with an energy width of 0.002 Ry to enhance self-consistent convergence. All simulations are conducted using periodic boundary conditions. We employ a supercell approach to minimize the elastic interactions between defects in periodic replica, with defective supercells containing one point defect at a time. The size of supercells is sufficiently large to impose a convergence of the vacancy formation enthalpies better than 0.02 eV to avoid the need of elastic energy corrections. We use defect-free supercells containing 3 × 3 × 3 conventional cells of fcc (108 atoms) and 4 × 4 × 4 of bcc (128) iron. An orthorhombic supercell (108 atoms) was constructed out of the primitive cell of hcp iron. Structure relaxation of perfect and defective supercells were performed at constant volume (*V*) with a large Brillouin zone *k*-point sampling on a 4 × 4 × 4 Monkhorst-Pack grid^57^ for fcc and bcc Fe and a 6 × 4 × 4 Monkhorst-Pack grid for hcp Fe to obtain full convergence of the electronic configurations until residual forces were minimized below 1.0 × 10^−5^ Ry/au. Further increase in supercell size did not significantly affect vacancy formation energies. Spin polarization is only considered for bcc iron (all pressures) and fcc iron at 0 GPa, since nonmagnetic states become energetically favorable with increasing pressure^26^. We find that the effect of spin polarization on the defect energetics in close-packed iron becomes insignificant above ~30 GPa.

### Defect energetics

Total energy calculations are conducted based on the constant pressure approach, so that total enthalpy is of concern. The point defect formation enthalpy is generally derived as

7 $$\Delta {H}_{f}=H(N\pm 1)-H(N)\frac{N\pm 1}{N},$$

where the negative and positive sign corresponds to vacancy and interstitial formation, respectively, *H*(*N*) is the enthalpy of a defect-free supercell containing *N* atoms and *H*(*N* ± 1) is the enthalpy of a supercell containing a single point defect.

The energy barrier of vacancy migration Δ*H*_*m*_ is defined as the enthalpy difference between its equilibrium (*H*_*eq*_) and transition state (*H*_*sp*_), when the migrating atom is located at its saddle point as

8 $$\Delta {H}_{m}={H}_{sp}-{H}_{eq}$$

In simple metallic systems such as fcc, bcc and hcp iron, saddle point configurations are constrained by the crystal symmetry to the middle between two nearest neighbor half-vacancies. Because of the lattice symmetry in bcc and fcc Fe, *H*_*sp*_ can be obtained by unconstrained structure relaxation of transition states. In the hcp phase, at least two atoms far from the vacancy need to be constrained during structure optimization. To verify the outcome of this approach, we performed CI-NEB calculations^27^ to find the minimum energy path (MEP) and the corresponding energy barrier of migration. The MEP is sampled using 7 images bonded by springs relying on the variable elastic constants scheme implemented in the Quantum ESPRESSO package. The initial and final configurations correspond to fully relaxed defective supercells with a vacancy at its equilibrium lattice site. Force minimization relies on linear interpolation between the initial and final configurations until the modulus of the force orthogonal to the path becomes less than 0.02 eV/Å. CI-NEB calculations are performed using a constant volume approach with the MEP obtained in terms of internal energy.

### Thermodynamic properties

Thermodynamic properties of the Fe systems are determined in the framework of lattice dynamics (LD) and electronic structure theory combined with the quasi-harmonic approximation (QHA). The LD calculations are performed based on the direct force constant approach^28^. Phonon frequencies *v*_*i*_ of supercells are computed by diagonalization of dynamical matrices using the PHONOPY code^58^ where force constants are generated using the finite displacement method. Atomic forces are determined via electronic structure calculations of relaxed supercells with displacements of 0.01 Å applied to all atoms around their equilibrium positions. Since vacancies break the original lattice symmetry, defective supercells of hcp and fcc Fe (107 atoms) require up to 642 displacements to build a single force constant matrix.

The EoS (Supplementary Fig. 2) and other thermodynamic properties (Supplementary Fig. 3) of hcp and fcc Fe are derived from the Helmholtz free energy (Eq. 3) using standard thermodynamic relationships (*e.g*. Tsuchiya^59^). The vibrational contribution to the Helmholtz free energy *F* is computed as

9 $${F}_{vib}(V,T)=\frac{1}{2}{\sum }_{q,i}h{\nu }_{i}(q,V)+{k}_{b}T{\sum }_{q,i}\mathrm{ln}\left(1-\exp \left(-\frac{h{\nu }_{i}(q,V)}{{k}_{b}T}\right)\right)$$

The contribution of Eq. 9 was evaluated on a 10 × 10 × 10 and a 12 × 10 × 10 **q**-grid for fcc and hcp Fe, respectively. For defective systems, Δ*S*_*conf*_ is approximated by the configurational entropy *S*_*conf*_ of an ideal solution with vacancy concentration *X* as

10 $${S}_{conf}=-{k}_{b}[X\,\mathrm{ln}\,X+(1-X)\mathrm{ln}(1-X)]$$

The electronic contributions to the free energy are evaluated as

11 $${F}_{el}={\sum }_{i}f({\varepsilon }_{i},T){\varepsilon }_{i}-T{S}_{el},$$

with the electronic entropy given by

12 $${S}_{el}=-\gamma {k}_{b}{\sum }_{i}[f({\varepsilon }_{i},T)\mathrm{ln}\,f({\varepsilon }_{i},T)+(1-f({\varepsilon }_{i},T))\mathrm{ln}(1-f({\varepsilon }_{i},T))],$$

where *γ* is 1 or 2 for spin polarized or unpolarized systems, respectively. The Fermi-Dirac distributions *f*_*i*_ are computed as function of the energies *ε*_*i*_ from the electronic density of states (eDoS). The magnetic contribution *S*_*mag*_ to the total entropy is evaluated as

13 $${S}_{mag}={k}_{b}\,\mathrm{ln}[m(2S+1)],$$

with total spin quantum number *S* = 2 and orbital degeneracy *m* = 3.

### Dislocation creep: the Weertman model

Weertman creep assumes that the glide velocity *v*_*g*_ of dislocations exceeds the velocity of dislocation climb *v*_*c*_ at high homologous temperature (*v*_*g*_ > *v*_*c*_) such as close to melting^37^. The average dislocation velocity *v* can then be approximated by *v* = *L*/*t*_*c*_, where *L* is the dislocation line length and *t*_*c*_ = *d*/*v*_*c*_ the time needed to climb a distance *d* between the glide planes. Assuming that the rate of strain $\dot{\varepsilon }$ produced by creep can be described in terms of Orowan’s equation $\dot{\varepsilon }={\rho }_{m}bv$, where *ρ*_*m*_ is the density of mobile dislocations and *b* the modulus of the Burgers vector, Weertman’s constitutive law can be easily derived as

14 $$\dot{\varepsilon }={\rho }_{m}b\frac{L}{d}{v}_{c}$$

The dislocation length *L* typically scales with the total dislocation density *ρ*_*t*_ as $1/\sqrt{{\rho }_{t}}$. We assume that all dislocations are partially mobile close to *T*_*m*_, *i.e. ρ*_*t*_ = *ρ*_*m*_. The climb velocity *v*_*c*_ can be represented by^60^

15 $${v}_{c}={A}_{c}\frac{{D}_{sd}}{b}\left[\exp \left(\frac{\sigma {V}_{A}}{{k}_{b}T}\right)-\frac{{X}_{\infty }}{X}\right],$$

where *A*_*c*_ is a geometrical factor and *X* and *X*_*∞*_ are the equilibrium vacancy concentrations in the bulk and far from the dislocation lines, respectively. The vacancy concentration far from the dislocation is supposed to be equal to that of the bulk (*X*_*∞*_ = *X*), given a cylindrical dislocation geometry ${A}_{c}=2\pi /\,\mathrm{ln}(1/2\sqrt{{\rho }_{t}}{r}_{c})$ and a dislocation core radius *r*_*c*_, typically ~10 Å. Based on the line tension, we use the Taylor relationship ${\rho }_{m}={(\sigma /\alpha \mu b)}^{2}$ to relate the mobile dislocation density to the applied stress^61^ and obtain the constitutive Eq. 5 in the limit of low stress, where $A={A}_{c}L{V}_{A}/{\alpha }^{2}{b}^{3}d$, and *α* a dimensionless parameter equal to ~0.1 under the assumption that the obstacle strength is predominantly governed by dipole interactions^62^. We like to note that the steady-state dislocation creep behavior derived here applies to the limiting case of high homologous temperature and low stress corresponding to the conditions of the inner core. Different temperature and stress conditions might affect the dislocation multiplication and annihilation processes, leading to the development of other microstructures, giving rise to different stress exponents.

## Supplementary information

Supplementary Information.

Supplementary Information 2.
