# Supplementary material for: Viscosity of hcp iron at Earth’s inner core conditions from density functional theory
Source: Sci Rep. 2020 Apr 14;10:6311. doi: 10.1038/s41598-020-63166-6 (PMC7156496; doi:10.1038/s41598-020-63166-6)
Supplement: Supplementary file 1 — Supplementary Information. [file 41598_2020_63166_MOESM1_ESM.pdf]

SUPPLEMENTARY MATERIAL OF THE PAPER:

Viscosity of hcp iron at Earth's inner core conditions from density functional theory

*Sebastian Ritterbex[1] and Taku Tsuchiya[1]

[1]Geodynamics Research Center, Ehime University, 2-5 Bunkyo-cho, Matsuyama 790-8577, Japan

Submitted to Scientific Reports

Corresponding author: *Sebastian Ritterbex

Affiliation: Geodynamics Research Center

Ehime University

2-5 Bunkyo-cho, Matsuyama

Ehime 790-8577, Japan

Tel: +81 89 927 8256

E-mail: ritterbex.sebastian_arthur_willem.us@ehime-u.ac.jp

Climbing image nudged elastic band (CI-NEB) calculations

To determine the minimum energy path (MEP) of out-basal plane vacancy diffusion in hcp Fe at 320 GPa, we performed CI-NEB calculations as described in the method section of the main paper. CI-NEB calculations are performed under the constant volume condition, where the MEP is obtained in terms of internal energy. The transition state was found exactly half-way between two nearest neighbor half-vacancies. The results are shown in Supplementary Fig. 1.

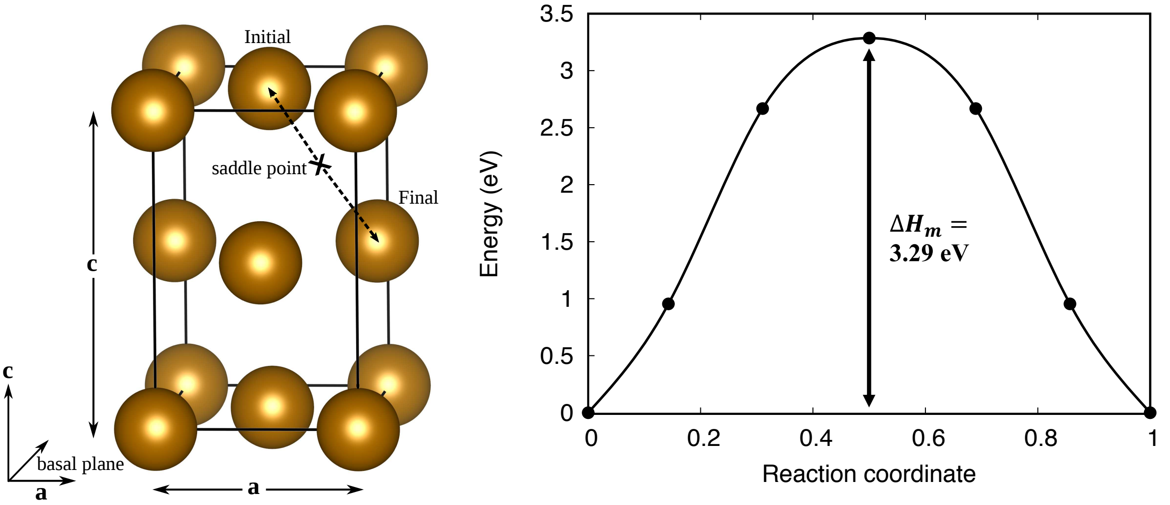

Supplementary figure 1. Atomic migration of out-basal plane self-diffusion in hcp Fe at 320 GPa and static temperature. The left-hand side illustrates the migration path of an Fe atom along the *c* direction between the basal planes. The structure corresponds to an orthogonal unit cell constructed out of a primitive hcp cell. From this orthogonal cell, a 108-atom supercell was prepared, in which the vacancy-vacancy interaction across the periodic boundary condition is almost negligible, and used to compute the MEP. The right-hand side shows the associated energy barrier of the MEP as calculated by the CI-NEB. The saddle point configuration corresponds to the maximum of the energy barrier, defined by the migration enthalpy ΔH_m as the energy difference between the transition and the initial/final states.

Thermodynamic properties

The phonon properties of defect-free and defective supercells are computed in the framework of standard lattice dynamics theory (see main text). The phonon frequencies of the

systems are obtained using the direct force constant method[1] by diagonalization of the dynamical matrix. Interatomic force constants were determined via electronic structure calculations of relaxed supercells with displacements of 0.01 Å applied to all atoms around their equilibrium positions along all the x, y and z directions. We verified that the phonon dispersion of a 108-atom supercell of defect-free hcp Fe computed within the direct method agrees with results obtained with the density functional perturbation theory[2] (DFPT).

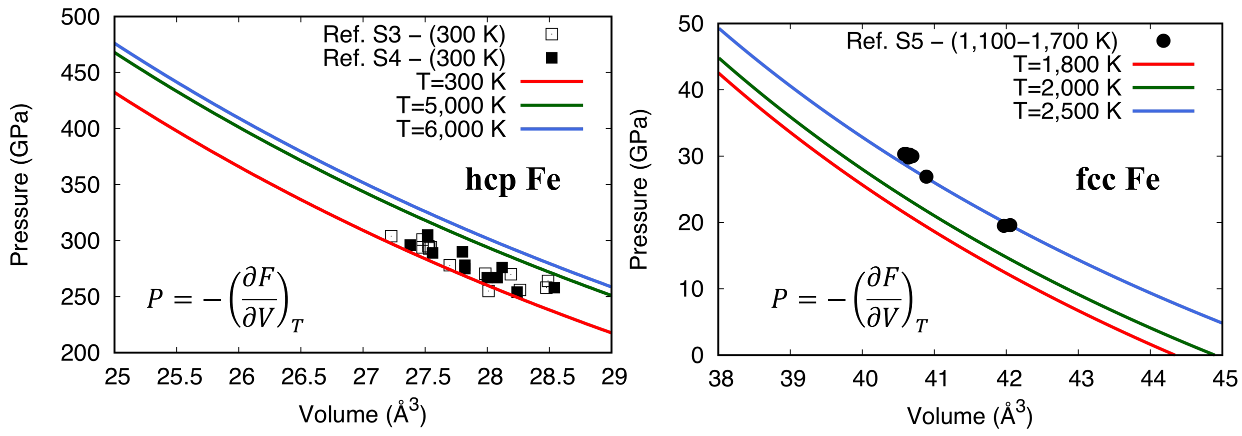

Supplementary figure 2. P-V-T equations of state (EoS) from free energy calculations. The P-V-T EoS are derived from the Helmholtz free energy variations as a function of volume fitted with a third-order Birch-Murnaghan[12] EoS. The total pressure P is given by the sum of the static P_{st} and thermal P_{th} contributions. Previous experimental results for hcp[3,4] and fcc[5] Fe are plotted for comparison.

The EoS and other thermodynamic properties of hcp and fcc Fe are computed using lattice dynamics (LD) theory and electronic structure theory in the framework of the quasi-harmonic approximation (QHA) (Supplementary Fig. 2 and 3). These properties are derived from the Helmholtz free energy (Eq. 3). The present results are in fair agreement with previous data (Supplementary Fig. 2 and 3). Experimental EoS of hcp Fe[3,4] are obtained at 300 K and are in good agreement with our results. Data for the fcc Fe[5] fairly agree with our results, although QHA-volumes are slightly underestimated at the same P, T. This might be related to spin polarization (Curie temperature) and technical difficulty of experiments at elevated temperature.

Based on the EoS of hcp and fcc Fe, the phonon frequencies of defective systems are computed. The phonon dispersion curves of defect-free and defective systems with a vacancy located at its equilibrium position are displayed in Supplementary Fig. 4.

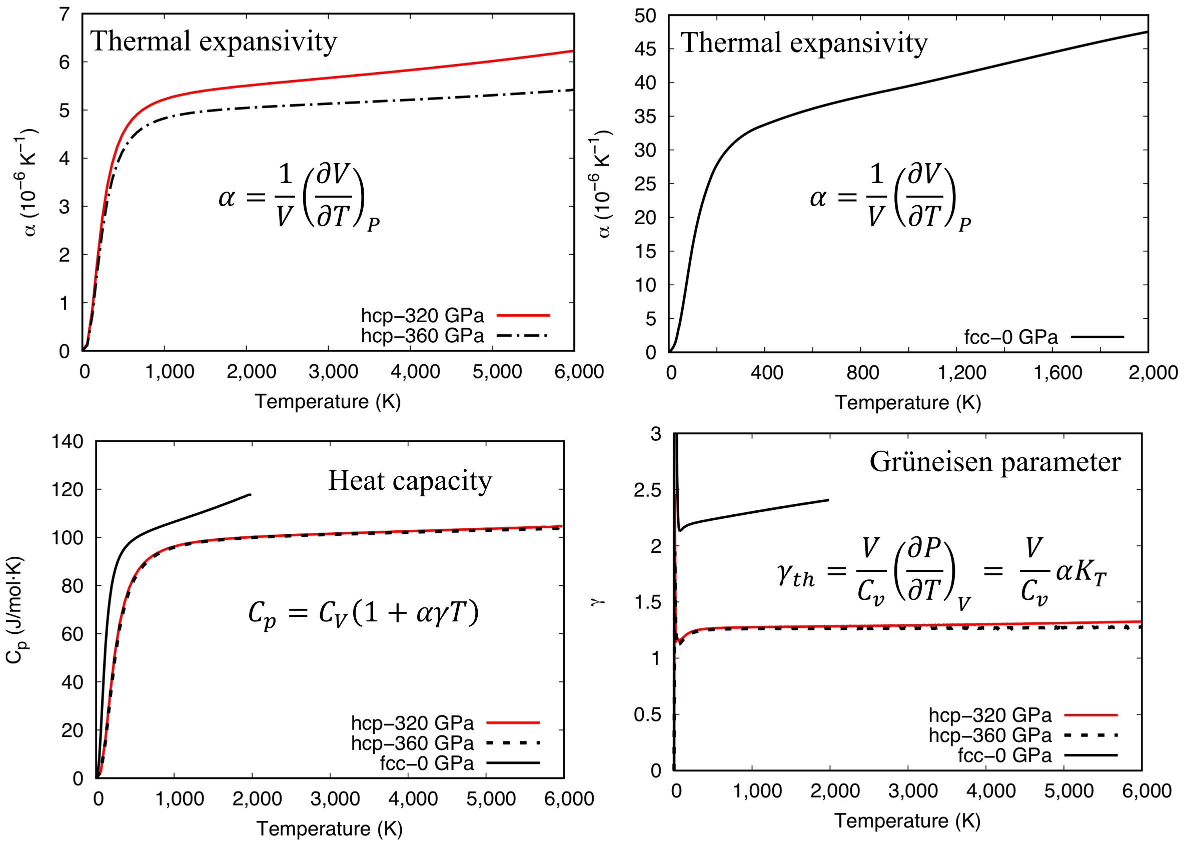

Supplementary figure 3. Thermodynamic properties of fcc and hcp Fe in the framework of the QHA. The thermal expansivity α, heat capacity C_p and Grüneisen parameter γ_{th} are represented as a function of T at fixed P. The Grüneisen parameter of hcp Fe ($\gamma = 1.3$) is in reasonable agreement with previous experiments[4,6], supporting small higher-order anharmonic effects.

The phonon frequencies are then used to compute the Helmholtz and Gibbs free energy of vacancy formation as explained in the method section of the main text. As discussed in Mantina *et al.*[7], differences in phonon dispersion between defect-free and defective systems qualifies the effect of entropy on the Gibbs free energy of vacancy formation. It can be observed from Supplementary Fig. 4 that the low-frequency portion of the vibrational density of states (DoS) of defective Fe shifts toward lower frequencies. This is due to the softening of bonds around the vacancy and yields a positive contribution to the formation entropy. On the other hand, the high-frequency part of the DoS of defective systems shifts towards higher frequencies, which is due to shorter Fe-Fe bond lengths observed in the vicinity of the vacancy. The latter is expected to cause a negative contribution to the formation entropy. From the vibrational density

of states (Supplementary Fig. 4), one can see that the low-energy shifts are more important, in agreement with the positive formation entropies obtained for both fcc ($7.35k_b$ at 0 GPa and 1,800 K) and hcp Fe ($3.26k_b$ at 360 GPa and 5,000 K). These significant entropies of vacancy formation must be considered to evaluate the vacancy formation energies appropriately.

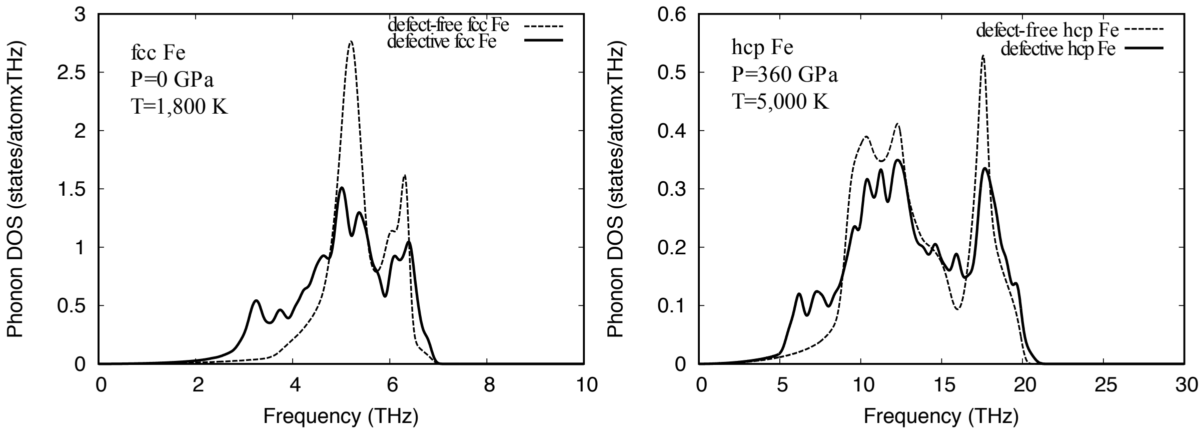

Supplementary figure 4. Vibrational density of states (DoS) of defect-free and defective fcc and hcp Fe. The left-hand side shows the vibrational DoS for defect-free fcc Fe (dotted line) and a system with a vacancy located at its equilibrium site (solid line). The right-hand side represents the same curves for hcp Fe.

The attempt frequency ν^* can be obtained in the framework of TST[8] as a function of the phonon frequencies of the equilibrium and transition states and is commonly close to ~10 THz in most metals. Supplementary Fig. 4 shows that the maximum phonon frequencies of defect-free and defective systems are very comparable. Because of the high computational cost of phonon spectrum calculations, particularly of transition states, and its small sensitivity to the final diffusivities, the attempt frequencies are estimated directly from the maximum frequencies of the phonon spectra following previous theoretical work[9].

Creep by diffusion of matter

The deformation mechanism responsible for plastic strain by pure atomic diffusion is commonly referred to as *diffusion creep*[10]. Diffusion creep is a grain size sensitive deformation mechanism and will be particularly activated when grain boundaries are the main sources and sinks of point defect (*i.e.* fine-grained materials). Nabarro-Herring creep corresponds to plastic

deformation by diffusional transport through the bulk of grains and is described by the following constitutive equation[10]

$$\dot{\varepsilon}_{NH} = A_{NH} \frac{D_{sd}\sigma V_A}{d^2\, k_b T} \tag{S1}$$

where d is the average grain size, $\dot{\varepsilon}$ the strain rate, σ the flow stress, V_A the activation volume of bulk diffusion, k_b the Boltzmann constant, and A_{NH} a shape factor frequently considered to be 16/3 (for a spherical grain geometry and where grain boundary sliding (GBS) is not allowed to occur)[10]. A deformation mechanism map for Nabarro-Herring creep of hcp Fe is presented in Supplementary Fig. 5, based on the parameters determined in this study (Fig. 1 and 2 of the main text), with viscosity $\eta = \sigma/2\dot{\varepsilon}$. Although the mean grain size in the inner core is essentially unknown, Bergman[11] provides arguments that the size of grains should be at least in the order of meters, leading to a high viscosity of ~10^{26} Pa s. Even a grain size in the order of millimeters leads to a viscosity larger then 10^{20} Pa s. These high viscosities rule out diffusion creep as an efficient strain producing mechanism in the inner core.

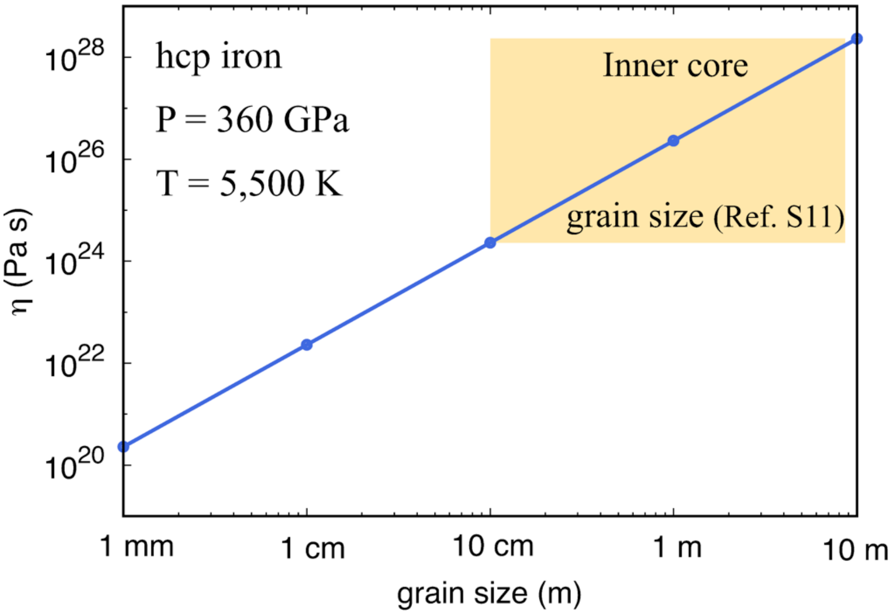

Supplementary figure 5. Deformation mechanism map for Nabarro-Herring creep of hcp Fe at inner core conditions. Results are shown by the viscosity η as a function of grain size d calculated based on the corresponding constitutive equation (SEq. 1) with the parameters obtained in this study. Bergman[11] provides some arguments that grains in the inner core are expected to have a size of ~1 m, resulting in very high viscosity values of ~10^{26} Pa s.

Supplementary references

[1] Wei, S. & Chou, M. Y. *Ab Initio* Calculation of Force Constants and Full Phonon Dispersion. *Phys. Rev. Lett.* **69**, 2799-2802 (1992).

[2] Tsuchiya, J., Tsuchiya, T. & Wentzcovitch, R. M. Vibrational and thermodynamic properties of $MgSiO_3$ postperovksite. *J. Geophys. Res.* **110**, B02204 (2005).

[3] Mao, H. H., Wu, Y., Chen, L. C. & Shu, J. F. Static Compression of Iron to 300 GPa and $Fe_{0.8}Ni_{0.2}$ Alloy to 260 GPa: Implications for Composition of the Core. *J. Geophys. Res.* **95**, 21737-21742 (1990)

[4] Sakai, T., Takahashi, S., Nishitani, N., Mashino, I., Ohtani, E. & Hirao, N. Equation of state of pure iron and $Fe_{0.9}Ni_{0.1}$ alloy up to 3 Mbar. *Phys. Earth Planet. Int.* **228**, 114-126 (2014).

[5] Komabayashi, T. & Fei, Y. Internally consistent thermodynamic database for iron to the Earth's core conditions. *J. Geophys. Res.* **115**, B03202 (2010).

[6] Dewaele, A., Loubeyre, P., Occelli, F., Mezouar, M., Dorogokupets, P. I. & Torrent, M. Quasihydrostatic Equation of State of Iron above 2 Mbar. *Phys. Rev. Lett.* **97**, 215504 (2006).

[7] Mantina, M., Wang, Y., Arroyave, R., Chen, L. Q. & Liu, Z. K. First-Principles Calculations of Self-Diffusion Coefficients. *Phys. Rev. Lett.* **100**, 215901 (2008).

[8] Vineyard, G. H. Frequency factors and isotope effects in solid state rate processes. *J. Phys. Chem. Solids* **3**, 121-127 (1957).

[9] Runevall. O & Sandberg. N. Self-diffusion in MgO – a density functional study. *J. Phys. Condens. Matter* **23**, 345402 (2011).

[10] Poirier, J. P. *Creep of Crystals* (Cambridge University Press, 1986).

[11] Bergman, M. Estimates of the Earth's inner core grain size. *Geophys. Res. Lett.* **25**, 1593-1596 (1998).

[12] Murnaghan, F. D. The compressibility of media under extreme pressure. *Proc. Natl. Acad. Sci. USA* **30**, 244-247 (1944).
